# First Autonomous Bio-Optical Profiling Float in the Gulf of Mexico Reveals Dynamic Biogeochemistry in Deep Waters

**DOI:** 10.1371/journal.pone.0101658

**Published:** 2014-07-03

**Authors:** Rebecca E. Green, Amy S. Bower, Alexis Lugo-Fernández

**Affiliations:** 1 Environmental Studies Section, Bureau of Ocean Energy Management, New Orleans, Lousiana, United States of America; 2 Physical Oceanography Department, Woods Hole Oceanographic Institution, Woods Hole, Massachusetts, United States of America; University of California, Merced, United States of America

## Abstract

Profiling floats equipped with bio-optical sensors well complement ship-based and satellite ocean color measurements by providing highly-resolved time-series data on the vertical structure of biogeochemical processes in oceanic waters. This is the first study to employ an autonomous profiling (APEX) float in the Gulf of Mexico for measuring spatiotemporal variability in bio-optics and hydrography. During the 17-month deployment (July 2011 to December 2012), the float mission collected profiles of temperature, salinity, chlorophyll fluorescence, particulate backscattering (*b_bp_*), and colored dissolved organic matter (CDOM) fluorescence from the ocean surface to a depth of 1,500 m. Biogeochemical variability was characterized by distinct depth trends and local “hot spots”, including impacts from mesoscale processes associated with each of the water masses sampled, from ambient deep waters over the Florida Plain, into the Loop Current, up the Florida Canyon, and eventually into the Florida Straits. A deep chlorophyll maximum (DCM) occurred between 30 and 120 m, with the DCM depth significantly related to the unique density layer ρ = 1023.6 (*R^2^* = 0.62). Particulate backscattering, *b_bp_*, demonstrated multiple peaks throughout the water column, including from phytoplankton, deep scattering layers, and resuspension. The bio-optical relationship developed between *b_bp_* and chlorophyll (*R^2^* = 0.49) was compared to a global relationship and could significantly improve regional ocean-color algorithms. Photooxidation and autochthonous production contributed to CDOM distributions in the upper water column, whereas in deep water, CDOM behaved as a semi-conservative tracer of water masses, demonstrating a tight relationship with density (*R^2^* = 0.87). In the wake of the Deepwater Horizon oil spill, this research lends support to the use of autonomous drifting profilers as a powerful tool for consideration in the design of an expanded and integrated observing network for the Gulf of Mexico.

## Introduction

Long-term monitoring of carbon cycling in the oceans is required to understand oceanic ecosystem response to natural and anthropogenic perturbations, including distinguishing trends due to storm events, climate cycles, oil spills, and global warming. Ocean primary production, as largely contributed to by phytoplanktonic carbon fixation, accounts for approximately half of the global estimated net primary production, an amount roughly equivalent to that on land [1]. This primary production represents the base of the marine food web, supporting nearly all oceanic life and significantly affecting global biogeochemical cycles, including atmospheric CO_2_ uptake. Thus, significant changes in phytoplankton biomass as linked to perturbations, such as climate forcing [2]–[3], can have major implications for marine ecosystem functioning all the way up the food chain. Photosynthetic phytoplankton are also largely responsible for the production of oceanic dissolved organic matter (DOM), which serves as substrate for heterotrophic microbial populations and provides nutrients for autotrophs. Marine DOM represents the largest oceanic pool of reduced carbon, estimated to hold greater than 200 times the carbon inventory of marine biomass [4]. Given the importance of both the marine particulate and dissolved organic matter pools, improved methods are required for jointly assessing and monitoring long-term changes due to perturbations, especially given linkages between the two components.

Autonomous profiling floats represent an emerging capability for monitoring biogeochemical properties of the world’s oceans at unprecedented scales. Technological advances in float platforms and sensor technologies allow deployments of longer duration (≥5 years), to greater depths (up to 2,000 m), and with higher sampling frequencies as smaller, lower-power sensors are developed [5]. Optical instrumentation recently developed specifically for float applications allows measurement of a suite of biogeochemical parameters, including concentrations of chlorophyll, particulate matter, colored dissolved organic matter (CDOM), dissolved oxygen, and nutrients. Recent miniaturization of sensors now allows for joint measurement of multiple parameters from floats, providing the ability to collect important baseline measurements and repeat monitoring for assessing long-term trends, such as climate-related impacts on ocean productivity, carbon cycling, oxygenation, and acidification [3]. Thus far, optically-equipped floats have been successfully used to provide broad spatial (horizontal and vertical scales) and highly time-resolved measurements of particle types and fluxes, including in the Pacific, Atlantic, and Mediterranean oceans [6]–[8], providing information on optical variability at previously unobserved scales. However, there are fewer examples of the simultaneous measurement of both particulate and dissolved organic matter cycling from floats, given only recent advances in the technology [9].

Deep waters of the Gulf of Mexico (GOM) represent an important frontier for better characterizing biogeochemical processes using autonomous platform technologies. Deep GOM waters provide valuable ecosystem services, including essential habitats for large pelagic species, deep sea corals, and marine mammals [10]. However, these waters are also heavily utilized by various industries, including commercial fisheries, shipping, and oil and gas production, with potentially harmful effects on the environment, such as the Deepwater Horizon oil spill. Previous work has suggested that a variety of environmental forcing factors can influence biogeochemical cycling in these deep waters, including seasonal mixing, Loop Current (LC) and eddy interactions, distant transport of riverine waters, and upwelling along the shelf edge [11]–[13]. However, thus far, an understanding of biogeochemical processes in deep GOM waters has been mostly limited to traditional shipboard sampling techniques and remote sensing studies of surface waters. More highly resolved sampling, in both time and 3-D space, is required to tease apart the various processes driving optical variability due to phytoplankton, particulates, and CDOM. To our knowledge, this is the first publication to describe the high-resolution measurements obtained from a bio-optical profiling float in deep waters of the Gulf of Mexico, including collection of both particulate and dissolved organic matter properties.

## Methods

**Figure 1:**
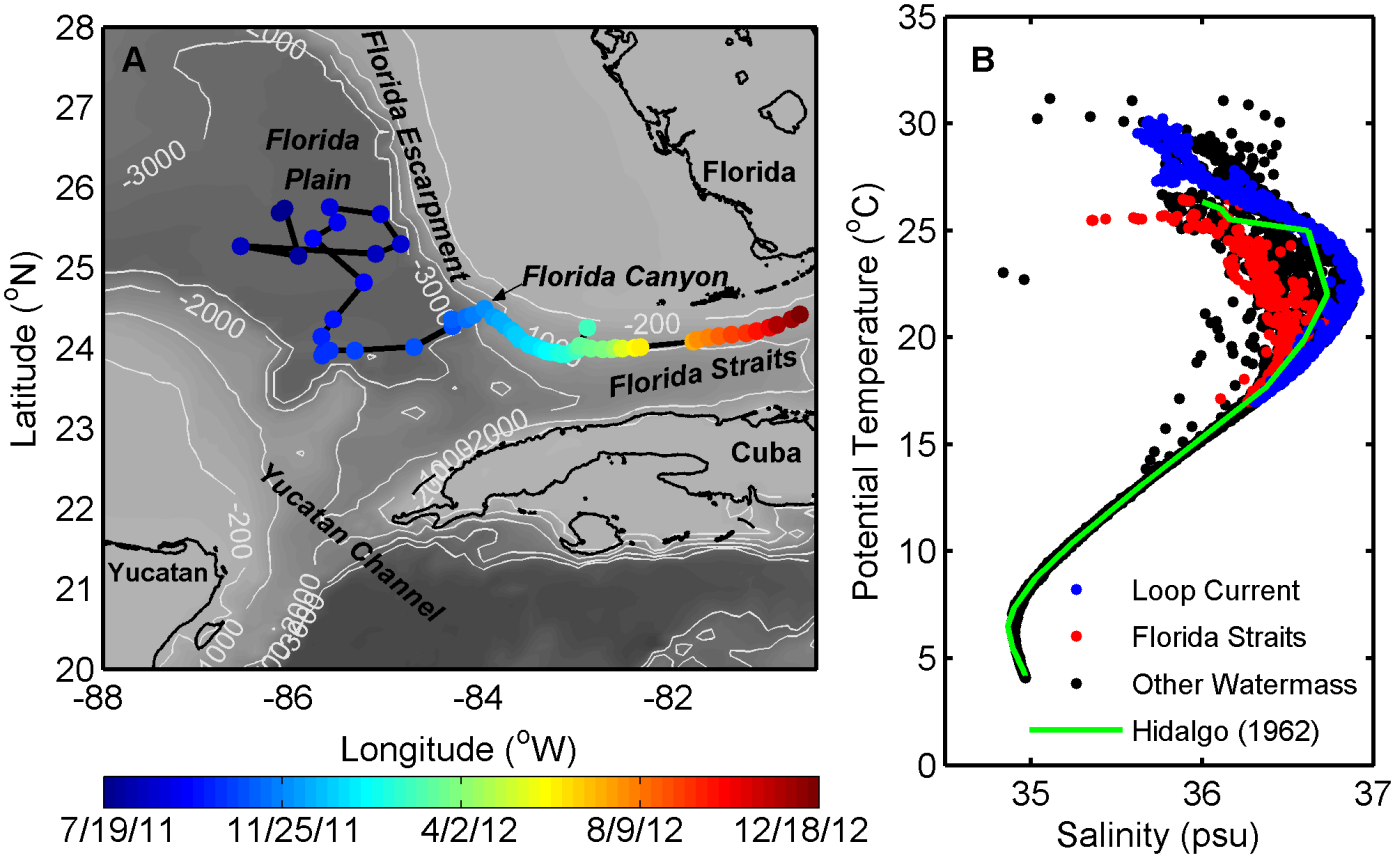
Map of float surface position in the Gulf of Mexico and T-S diagram for the deployment. (A) Float surface position, starting with its deployment on July 19^th^, 2011 and showing each surfacing (circles), until its last useful profile on December 18^th^, 2012. Note that profiles are not evenly spaced in time (see Methods). (B) T-S diagram for the float deployment demonstrating characteristic shape for the GOM, including profiles associated with the LC, Florida Straits, and all other water masses sampled. The T-S relationship compares well with an example profile from the historic Hidalgo (1962) cruise.

The measurements presented here were collected as part of the Bureau of Ocean Energy Management (BOEM)-funded “Lagrangian Study of the Deep Circulation in the Gulf of Mexico”, which is measuring currents at depth in both U.S. and Mexican waters. In totality, the study has deployed ∼120 acoustically-tracked RAFOS floats [14] at depths of 1500–2500 m to map the deep circulation and its variability, as well as 8 autonomous profiling APEX floats [15], the majority of which (at the time of writing this paper) are still collecting measurements. However, one of the APEX floats has now finished its mission, after 17 months of deployment, and is the topic of this paper (Fig. 1A; Dataset S1). The deployment of the profiling float for this study did not require permits for the following reasons: 1) it was deployed in federal waters of the US or inside the Exclusive Economic Zone and not in State waters, and 2) it was deployed under a study for the BOEM of the US Dept. of the Interior, under the authority of the Outer Continental Shelf Lands Act. This Act requires the Agency to conduct studies to evaluate the potential impacts of the oil and gas industry on the environment.

The APEX float was equipped to provide profiles of both physical and bio-optical measurements. The profiling float was built by Teledyne-Webb Research, Inc. (with float dimensions of 16.5 cm diameter by 127 cm long; Fig. S1) and was interfaced with a pumped conductivity-temperature-depth (CTD) instrument (SBE41-CP, SeaBird), bio-optical sensors (ECO FLbbCD-AP2, WET Labs, Inc.), and two-way Iridium communications, which allowed for both real-time data transmission and sampling plan adjustments. The instrument was controlled to float at a specified depth and to profile at set time intervals throughout the water column. While the profiler was at its specified park depth, it acted as a passive, quasi-Lagrangian current follower. The vertical resolution of float bio-optical sampling was set to provide increasing resolution towards the surface, as follows: 5 m from 0–200 m water depth, 10 m from 200–500 m water depth, 25 m from 500–1,000 m water depth, and 50 m below 1,000 m water depth.

The float profiled in the southeastern Gulf of Mexico, traveling from deep waters of the Florida Plain, along the West Florida Escarpment, and into the Florida Straits (Fig. 1A). It collected a total of 61 water column profiles (equaling 5,514 discrete measurements) of bio-optical and physical properties during a 17-month period. The float was deployed from the R/V Pelican on July 19^th^, 2011 over the Florida Plain, where the water depth was ∼3,200 m, and transmitted high-quality data through December 18^th^, 2012 when it left the GOM through the Florida Straits and was not retrieved. During its first 140 days of deployment (through Dec. 6^th^, 2011), the float collected measurements down to 1,500 m in deep GOM waters highly impacted by the LC. After traveling significantly to the southeast of its initial deployment, the float began moving into shallower waters of the West Florida Escarpment and up the Florida Canyon. As the float moved into shallower waters, it likely rested on the bottom between profiles. Then, as the float rose to the surface and descended again, it was advected by the currents, thus landing in a slightly different spot on the bottom. While profiling was initially set to every 14 days (7/19-8/17/2011), it was quickly decreased to every 5 days to upload data more often and clear out the memory backlog (8/17-4/9/2012). Finally, sampling was increased again to every 14 days in the Florida Straits to maximize float time sitting on the bottom, in order to delay its leaving the GOM (4/9-12/18/2012).

The bio-optical sensor suite on the float measured proxies of phytoplankton abundance (chlorophyll fluorescence), total particle concentration (optical backscattering), and dissolved organic matter (CDOM fluorescence). Calibration of the sensors (serial # FLBBCDAP2-2140) was performed by the manufacturer (WET Labs, Inc.) prior to shipping for installation on the floats. Dark counts were determined by the manufacturer using the signal output of the sensor in clean water with black tape over the detector. Sensor scaling factors were determined for each sensor using appropriate standards (i.e., a mono-culture of phytoplankton for chlorophyll fluorescence, microspherical beads for backscattering, and a quinine sulfate dihydrate solution for CDOM fluorescence). A separate field characterization was not performed on the sensors and thus, it is possible that some variation from the dark counts and scale factors determined by the manufacturer may have occurred due to factors in the field. Data from each of the sensors, output in counts, was converted to engineering units using the laboratory calibrations, resulting in chlorophyll concentration (Chl; µg l^−1^), the volume scattering function at a centroid angle of 140° and a wavelength of 700 nm (*β*(140°, 700 nm); m^−1^ sr^−1^), and CDOM concentration (ppb). Volume scattering data contained significant spikes (perhaps associated with particulate aggregates), and the data was despiked by applying a 3-point running minimum filter followed by a 3-point running maximum filter to separate spikes from the baseline, similar to Briggs et al. [16]. The volume scattering function of seawater, *β_sw_*(140°, 700 nm) was calculated following Zhang et al. [17] and subtracted from *β*(140°, 700 nm) to yield the scattering due to particles, *β_p_*(140°, 700 nm), which was converted to integrated particulate backscattering, *b_bp_*(700 nm), according to:
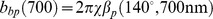
where χ = 1.132 for this optical configuration (James Sullivan [WET Labs, Inc.], personal communication). While the instruments were not re-calibrated during the deployment, sensor drift was considered to be relatively small given the observed temporal stability of sensor output in the field. For example, at a reference depth of 400 m where low variability was generally observed in optical properties, only small differences were observed between the first and second half of the deployment (differences for Chl, bbp, and CDOM of 0.006 µg l^−1^, 6×10^−6 ^m^−1^, and 0.01 ppb, respectively).

Analyses in silico were performed to describe observed variability in the bio-optical datasets, including comparison to the physical datasets collected. In addition to temperature (T) and salinity (S) measured by the float, an ancillary dataset of sea surface height anomalies (SSHA) was also employed. For the period corresponding to float deployment, SSHA fields were determined from remotely-sensed altimetric data obtained from the Colorado Center for Astrodynamics Research (CCAR, courtesy of Robert Leben). The criterion used for LC waters was defined as SSHA ≥17 cm [18]. All analyses of physical and bio-optical datasets, including creation of figures, were performed using the MATLAB software package (The MathWorks).

## Results and Discussion

**Figure 2:**
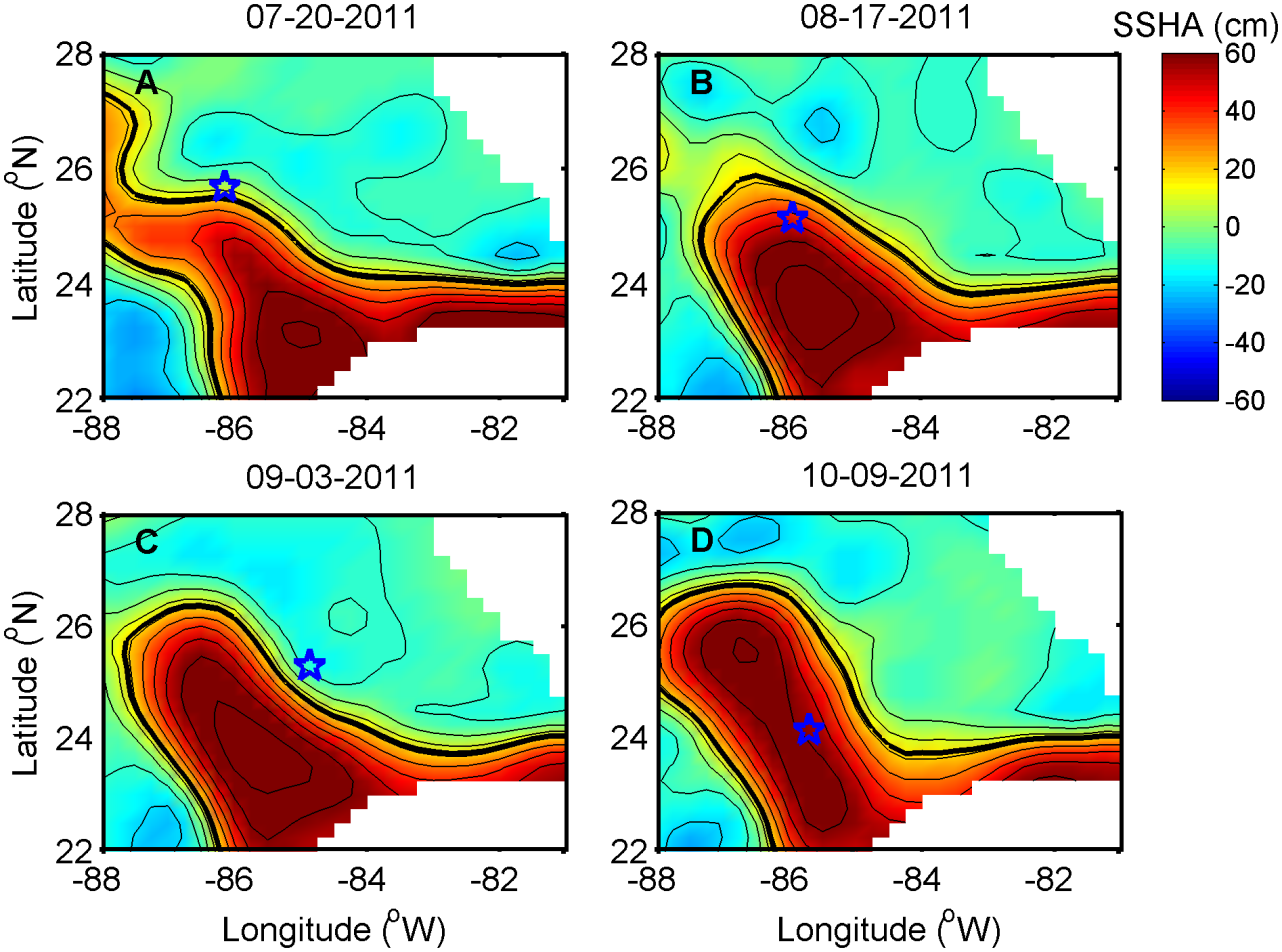
Example comparisons of SSHA fields to float surfacing locations on four dates. (A) July 20^th^, (B) August 17^th^, (C) September 3^rd^, and (D) October 9^th^, 2011. The float location (blue star) is identified relative to the Loop Current boundary, as determined by the 17-cm isopleth (thick black line).

Float physical and remote sensing data indicated the various unique water masses sampled, including Loop Current and Florida Straits waters. Over all float measurements, temperatures and salinities ranged between 4.2–31.2°C and 34.8–36.9 psu, respectively (Fig. 1B). Float temperature and salinity data compared well to historical data from the Hidalgo (1962) cruise in the GOM [19], showing the same distinctive T-S relationship. Profiles were often associated with the LC in the upper layer, especially over the Florida Plain, demonstrating unique T-S characteristics, which were distinct from other profiles. In particular, the LC was associated with the maximum temperatures sampled in surface waters, and generally defined the outer envelope of the T-S diagram (Fig. 1B). In contrast, waters in the Florida Straits typically did not retain the unique LC signature and rather defined the inner envelope of the T-S diagram, likely due to mixing with ambient waters (Fig. 1B). In the realm below ∼17°C, deeper waters had highly uniform physical properties, fitting a tight T-S relationship. The interaction of the float with the LC was also apparent through comparison with altimetry data, with several crossings of the LC boundary observed, especially during the first part of the deployment in deep waters over the Florida Plain (Fig. 2). Values of SSHA, matched up to float surfacings, ranged from a minimum of −14 cm on September 3^rd^, 2011 to a maximum of 58 cm on October 9^th^, 2011.

**Figure 3:**
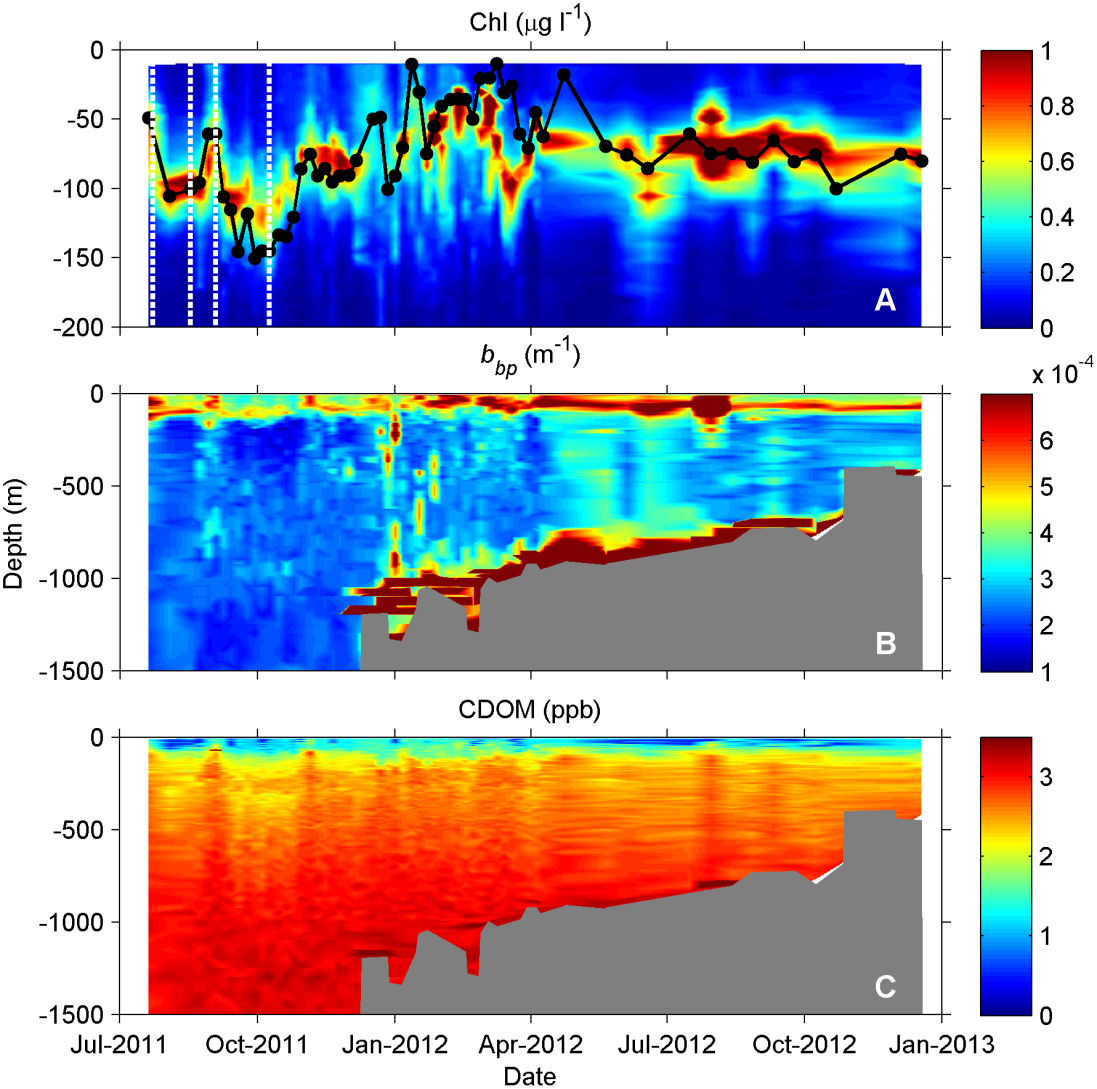
Contour plots demonstrating spatiotemporal variability in bio-optical float profiles. (A) Chl, (B) *b_bp_*, and (C) CDOM. Note difference in vertical axes for Chl (upper 200 m) versus *b_bp_* and CDOM (entire depth profile) to emphasize depth zones of maximum variability. For reference, the density layer ρ = 1023.6 is shown in panel A (black line) with sample times (black dots), and bottom depth is shown in panels B and C (shaded grey). The times corresponding to SSHA imagery in Fig. 2 are indicated in panel A (white dashed lines) to show float location relative to the LC boundary.

**Figure 4:**
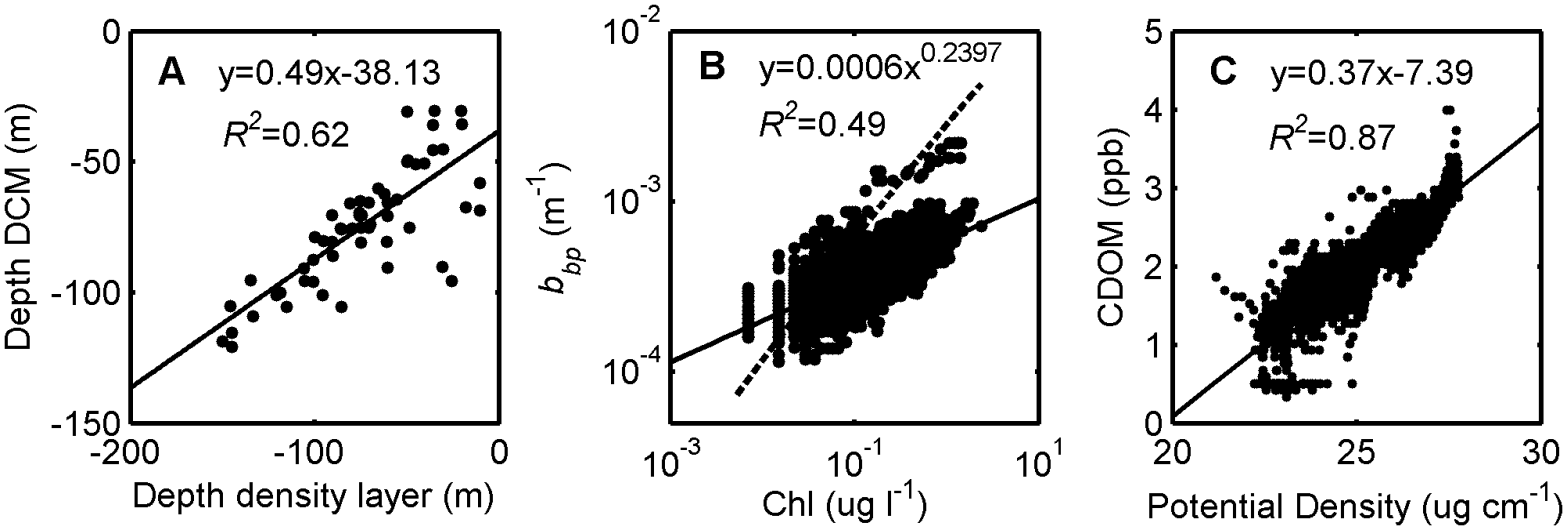
Bio-optical and bio-physical relationships determined based on float profile dataset. (A) depths of DCM vs. density layer ρ = 1023.6, (B) Chl vs. *b_bp_* in the upper 200 m, and (C) CDOM vs. potential density over the entire water column. In panel B, comparison is shown to results from the algorithm of Morel and Maritorena ([23], dashed line) [2001]. The anomalously high *b_bp_* values correspond to the float profile from July 30^th^, 2012, during which time a plume of terrigenous origin was advected offshore into the float’s path.

During the sampling period, variable chlorophyll concentrations contributed evidence supporting a heterogenous picture of deep Gulf of Mexico oligotrophic waters, as punctuated by spatial hot spots and temporal peaks in biomass. The majority of variability in Chl occurred in the upper water column (above 200 m; Fig. S2A) where concentrations ranged from 0.01 to 2.38 µg l^−1^. Average values observed near-surface (0.14±0.09 µg l^−1^; Fig. 3A) were similar to those previously reported in offshore GOM waters [13], [20]. However, the highly-resolved float measurements demonstrated a greater degree of variability in Chl, with concentrations in the Deep Chlorophyll Maximum (DCM) on average 10 times higher than at the surface, and as much as 30–40 times higher in some locations. The DCM ranged in depth from 30 to 120 m (Fig. 3A), with the average depth greater in deep GOM waters (91±18 m) versus in the Florida Straits (65±18 m). Notably, the depth of the DCM approximately doubled as the float twice moved from outside to inside the LC during the first five months of the deployment (Figs. 2, 3A), indicative of a deeper nitracline in the LC compared to ambient waters [13]. Pycnocline shoaling and resulting subsurface upwelling events offshore of the Southwest Florida Shelf punctuated surface Chl with relatively high values, such as in the deep GOM during 8/29-9/3/2011 and in the Florida Straits during 12/17/2011–2/12/2012 when surface Chl reached ∼0.3–0.4 µg l^−1^ (Fig. 3A). Across the entire deployment, DCM depth was highly correlated to the depth of the density layer ρ = 1023.6 (Fig. 4a; *R^2^* = 0.62, *p*<0.001), which corresponded to a mean temperature of 25.4°C and salinity of 36.4 psu and roughly to the depth of the pycnocline (Fig. S4).

**Figure 5:**
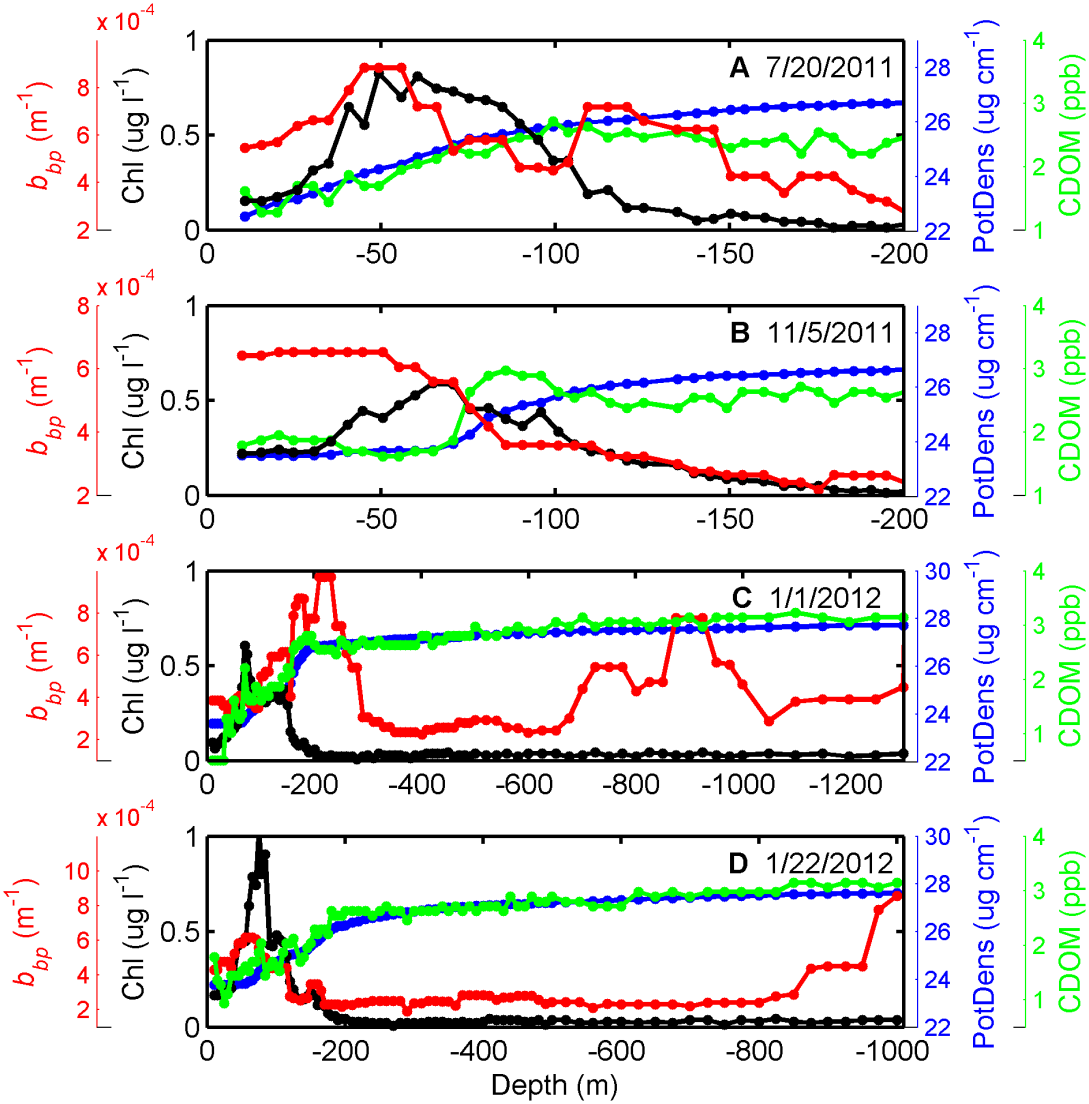
Float profiles exemplifying depth trends and peaks in *b_bp_*, Chl, CDOM, and potential density (PotDens). Examples each are provided of *b_bp_* peaks at the following depths: (A) coincident with and just below the DCM, (B) in a surface layer, (C) at intermediate depths (700 to 900 m), and (D) near-bottom. In panels A and B, only the upper 200 m of the profiles are shown to emphasize the region of maximum variability in *b_bp_*.

As an indicator of the total particulate pool, backscattering demonstrated significant complexity throughout the water column, with contributions from diverse particle types. Natural particle assemblages contain a range of living and non-living particles, which can all contribute to optical backscattering in the ocean, depending on their composition and size distribution, including phytoplankton, heterotrophic organisms (mostly bacteria), viruses, detritus, and minerals [21]–[22]. While backscattering often peaked with chlorophyll (Figs. 4b, S3), it also demonstrated high values at other depths in the water column, indicative of the unique dynamics of the total particulate pool (Fig. 3B). Across the entire float deployment, peak particle concentrations typically occurred at the following depths: (1) coincident with chlorophyll peaks in the upper layer (Fig. 5A), (2) just below the DCM (Fig. 5A), (3) in a surface layer (Fig. 5B), (4) at intermediate depths (200 to 1000 m; Fig. 5C), and (4) near-bottom (Fig. 5D). Elevated *b_bp_* values in the upper water column (above 200 m) often occurred at the same locations as high values of chlorophyll (Figs. 5A, S3A–B), as evidenced by the significant relationship between *b_bp_* and Chl (Fig. 4b; *R^2^* = 0.49, *p*<0.001). The smaller observed slope between *b_bp_* and Chl for most of the dataset (Chl>0.03 mg m^−3^) compared to other oceanic regimes (Fig. 4B, [23]), suggests a lower backscattering efficiency for phytoplankton and associated particles in the GOM due to differences in particle size distribution and/or composition. As indicated by ocean color imagery, anomalously high *b_bp_* on July 30^th^, 2012 (Fig. S3b, 4b) appears to have been linked to a plume of terrigenous origin advected offshore into the float’s path, introducing a water mass with a significantly different particulate and dissolved composition. Future studies analyzing individual particle characteristics, in addition to bulk optical properties [22], would contribute better understanding of the roles that distinct particle types and characteristics play in determining the optical field in deep GOM waters.

In the oligotrophic waters of the open Gulf of Mexico, the deep chlorophyll maximum and associated biological community play a significant ecological role in structuring the food web. Evidence presented here demonstrates consistently elevated chlorophyll concentrations at depth in the Southeastern GOM (Fig. 3A), and an associated particle assemblage as evidenced by high backscattering values (Fig. 4B). These hot spots of chlorophyll and related primary production occur where nutrient availability is locally enhanced, such as at the pycnocline depth, and are a significant contributor to water column primary production, recently estimated at a median value 0.28 gC m^−2 ^d^−1^ for the open Gulf [24]. On an areally-integrated basis, this median estimate resulted in the open Gulf having a larger regional primary production budget than the shallower Gulf regions (i.e., West Florida Shelf, Louisiana Shelf, Texas Shelf, and Mexican Shelf). In terms of the carbon pump, the net result of physical and biological factors in the open Gulf is such that it is also one of the largest net sinks of CO_2_ of all Gulf regions, estimated at −0.48 mol C m^−2^ y^−1^ [25]. The export of organic matter (marine snow) from this biological pump in the open GOM helps support a diverse benthic habitat of bacteria, meiofauna, megafauna, fishes, and deep water corals [26]–[27]. In addition to phytoplankton, productivity hot spots are also associated with higher stocks of zooplankton and micronekton in the deepwater GOM [20], [28]. Grazing of algal cells by zooplankton may be responsible for pheophytin peaks observed in previous studies just below the chlorophyll maxima, and thus, may explain the *b_bp_* peaks observed in the present study just below the DCM (Fig. 5A). Virus and bacterial concentrations can also be highly correlated with chlorophyll in the oligotrophic southeastern GOM [29]. Together, the association with phytoplankton of these diverse particle types likely contributed to the high backscattering signal observed in and around the DCM in the present work.

In the lower layer of the water column, backscattering often peaked in deep scattering layers (DSL) and demonstrated highest values near-bottom. The majority of profiles contained backscattering peaks in DSL between 200 to 1,000 m, with layer thickness ranging from 10′s to 100’s of meters (e.g., Figs. 3B, 5C). These layers may have contributions from a variety of sources, including aggregations of zooplankton and micronekton at depth, as previously observed using acoustics in other parts of the GOM [30], and/or horizontal advection of particles seaward from the continental slope [31]. Zooplankton and related particles are a likely explanation, especially for profiles in deeper waters away from the slope, and are indicative of a potential prey source for higher trophic levels, including cetaceans, which frequent these waters. Due presumably to contributions from non-living particles, the highest values of *b_bp_* occurred near the bottom as the float profiled into shallower waters and close to the maximum water depth (Fig. 3B, 5D), with values as high as 0.01–0.05 m^−1^. These high near-bottom *b_bp_* values are indicative of either natural resuspension in a bottom nepheloid layer or resuspension by the float itself landing on the bottom and disturbing the top sediment layer, while it sat at the bottom in shallower depths between profiles. However, the latter possibility of resuspension by the float landing would likely have been localized in space and would not explain the high backscattering observed 100’s of meters above the bottom (e.g., Fig. 5D). If natural resuspension is the cause, then these measurements indicate currents strong enough at the bottom, at depths of 700–1,200 m, to resuspend particulate matter. Such resuspension events in this region are plausible given the predominance of mud as a bottom type [32] and maximum near-bottom current speeds of 40–60 cm s^−1^ (unpublished data from BOEM “Loop Current Dynamics Study”). However, the true cause of high bottom backscattering would need to be further investigated in the future to remove the potential for sampling artifacts. Further bottom boundary layer experiments could also help elucidate environmentally-relevant mass (sediment) flows and net fluxes of resuspended materials. Based on previous research, it is most likely that the dense water of these particle-laden lower layers in the Florida Straits cannot pass through the shallower sections further downstream [33].

In the upper water column and near-bottom, vertical variability in CDOM profiles demonstrated the various biological and physical sources and sinks that can impact this optically-active dissolved organic matter pool, including autochthonous production, photobleaching, and resuspension. The fast turnover, most bioavailable forms of dissolved organic carbon occur in the surface ocean, in contrast to the longer-lived and more recalcitrant materials which circulate in deep oceanic waters [34]. In the open ocean, possible sources of CDOM production include excretion by organisms, viral lysis, and remineralization of sinking particulate matter, which variously contribute to both the deep CDOM reservoir and mixed layer CDOM; the major sink in the latter is photobleaching as controlled by irradiance and mixed layer depth [35]–[36]. Across all profiles in our dataset, CDOM was lowest in surface waters ranging from 0.3 to 1.9 ppb (Fig. 3c, S3c), with photobleaching as the major sink in the upper mixed layer, especially during the non-winter months when the water column was more stratified. Similarly, low CDOM has previously been reported in surface Sargasso Sea waters, where stratification and high solar radiation levels lead to bleaching countering local production of CDOM [37]. The high vertical resolution in our profiles did provide evidence of localized contributions from autochthonous production, with CDOM peaks in the upper layer corresponding to peaks in particles. For example, more than half of the profiles clearly demonstrated CDOM peaks co-occurring with elevated chlorophyll concentrations (e.g., Fig. 5B–D), and occasionally coinciding with *b_bp_* peaks as well. A steep increase in CDOM of 2–6x was observed between the surface and the pycnocline (Fig. S3c), with such increases also observed in the Sargasso Sea [37]. In our dataset, highest CDOM values were observed near-bottom, reaching a maximum of 4.0 ppb (Fig. 3C), presumably corresponding to resuspension events, though sampling artifacts again can not be ruled out. In New England shelf waters, Boss et al. [38] collected data supporting that bottom sediments can act as a source of dissolved organic carbon during sediment resuspension events. However, this is the first time measurements have suggested this phenomenon in deep GOM waters.

**Figure 6:**
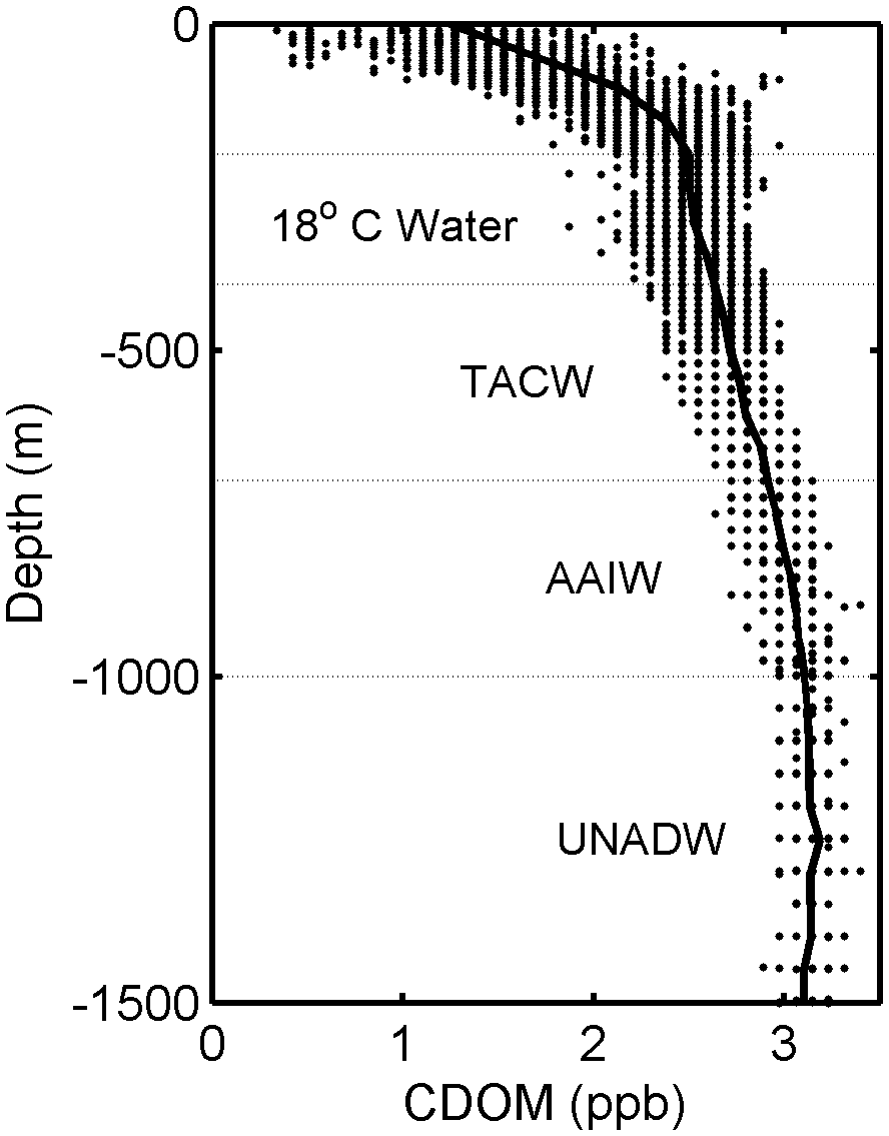
CDOM from all float profiles overlaid with the major Gulf of Mexico water masses below 200 m. The primary deep water masses include: 18°C Sargasso Sea water (200–400 m), Tropical Atlantic Central water (TACW, 400–700 m), Antarctic Intermediate Water (AAIW, 700–1,000 m), and Upper North Atlantic Deepwater (UNADW, ∼1,000 m and greater) [19]. Mean CDOM values for all profiles is indicated (solid black line). Note that all CDOM >3.5 ppb were removed from this figure and associated mean, to remove anomalies due to high near-bottom values.

At intermediate depths and greater, changes in CDOM were largely consistent with physical mixing and water mass distributions, suggesting its utility as a semi-conservative tracer at depth in the Gulf of Mexico. CDOM concentrations in deep GOM waters roughly followed the same depth patterns previously observed for nutrients, such as nitrate and phosphate [39], in each of the primary deep water masses, which include: 18°C Sargasso Sea water (depths 200–400 m), Tropical Atlantic Central water (TACW, depths 400–700 m), Antarctic Intermediate Water (AAIW, depths 700–1,000 m), and Upper North Atlantic Deepwater (UNADW, depths ∼1,000 m and greater) [19]. Below 200 m, CDOM continued to increase with depth to 1,000 m, though with a much smaller rate of change than in the upper water column; below 1,000 m, CDOM was approximately constant with depth (Fig. 6). Average CDOM concentrations equaled the following values in each of the water masses: 2.5 ppb in 18°C Sargasso Sea water, 2.7 ppb in TACW, 3.0 ppb in AAIW, and 3.1 ppb in UNADW. Below 200 m, CDOM concentrations were strongly and positively related to potential density (*R^2^* = 0.87, *p*<0.01; Fig. 4c) and temperature (*R^2^* = 0.81; not shown), indicating physical mixing as an important determinant of variability and the role of CDOM as a semi-conservative oceanographic tracer in GOM deep waters. Past studies in the North Atlantic have supported the potential of CDOM as a tracer of ocean circulation processes for subducted water masses [35], and our present results lend evidence for a similar role in the Gulf of Mexico.

During and following the Deepwater Horizon oil spill, a ship-based dataset of CDOM fluorescence was collected in the northcentral Gulf of Mexico in order to track the presence of the subsurface hydrocarbon plume. This dataset spanned the time period from just after the oil spill started until several months after the well was capped (May to October, 2010, [40]). While those CDOM profiles were generally in a similar range of values and demonstrated a similar depth increase to our dataset, many of the profiles showed large spikes in CDOM at depths of ∼800–1,200 m corresponding to the presence of the subsurface hydrocarbon plume [41]. However, as expected given the location of the present float dataset (≥400 km to southwest of spill site) and length of time since the oil spill (≥1 year), the deepwater hydrocarbon fluorescence anomaly evidenced in the oil spill dataset was not present in this float data. During future oil spill events, bio-optically equipped profiling floats could prove a useful tool for improved detection of subsurface hydrocarbon plumes, in addition to traditional ship-based measurements.

## Conclusions

We observed highly dynamic biogeochemistry in both the particulate and dissolved matter pools in deep waters of the southeastern Gulf of Mexico, using an APEX profiling float equipped with bio-optical sensors. Understanding such variability in the open ocean is important because the particulate and dissolved pools play a key role in determining underwater light availability and the resulting impact on biogeochemical cycling. As well, in deeper layers of the water column, bio-optical variability provides insight into the various oceanographic and biological processes at play. However, there are few previous examples of deepwater bio-optical studies in the Gulf of Mexico, in comparison to numerous studies in shallow and shelf regions [42]–[44]. The present study demonstrated complex variability in the particulate matter pool in deep GOM waters, as measured by chlorophyll fluorescence and optical backscattering (*b_b_* (700 nm)), with peaks observed at various depths throughout the water column. This dataset provided evidence for a dynamic DCM in the GOM impacted by mesoscale processes, as well as evidence for the formation of deep scattering layers between 200–1,000 m, likely of biological origin, and the potential importance of sediment resuspension at depths >500 m. As well, backscattering was significantly related to chlorophyll concentration in the upper water column (Fig. 4B), a parameterization which could improve ocean color, satellite-based retrievals of phytoplankton biomass, as it has in other oceanic regimes [45].

Additionally, the present study provided evidence of the important role that water column density structure, as impacted by water mass variability and vertical mixing, plays in structuring both particulate and dissolved concentrations in the deep GOM in addition to other processes (e.g., photo-oxidation, autochthonous production, grazing, etc.). While previous studies have suggested such a role in the GOM [28], the large number of observations afforded by the present float deployment allowed actual parameterization of the relationships of both chlorophyll and CDOM versus water column density (Fig. 4), including in the wintertime when ship-based measurements are typically rare. Such relationships could significantly improve the current emerging generation of Gulf-wide coupled, biogeochemical models, which aim to capture the spatiotemporal variability in particulate and dissolved matter pools [46]–[47]. For example, the present float measurements help fill an important deepwater gap in model validation data, with the predominance of data currently in shallow waters, such as the Louisiana-Texas shelf [47].

The emerging role of autonomous underwater vehicles (AUVs) promises to be a critical asset in future ocean observing systems, as they provide economical, long-term deployments and measurements at unprecedented resolution. As well, such autonomous platforms allow for sampling during high-wind periods, when traditional oceanographic methods are impracticable. The present study demonstrates the utility and feasibility of optically-equipped profiling floats for providing new understanding of biogeochemical processes in deep GOM waters, at a critical time for the future of ocean observing in this region. Following the Deepwater Horizon oil spill, in 2012 the U.S. Congress passed the RESTORE Act, which was created to invest oil spill funds into recovering GOM ecosystems that were affected by the disaster. Marine ecosystem monitoring is amongst the activities that can be funded by this legislation, with the oil spill having acutely demonstrated the need for improved oceanographic observing systems [48]. Looking towards the future, our research lends support to the use of autonomous drifting profilers as a powerful tool for consideration in the design of such an integrated observing network for the Gulf of Mexico.

## Supporting Information

Figure S1 **Picture of APEX float being deployed in the Gulf of Mexico.** The antenna and pumped CTD are located at the top of the float, whereas the optical sensors are located near the bottom of the instrument (Photo Credit: CANEK group, CICESE).
(TIF)

Figure S2 **Contour plots of bio-optical profiles over the entire depth range the float transited.** (A) Chl, (B) *b_bp_*, and (C) CDOM. Bottom depth is shown for reference (shaded grey). Note that profiles are not evenly spaced in time (see Methods).
(TIF)

Figure S3 **Contour plots of bio-optical profiles for the upper 200 m to emphasize upper-water column dynamics.** (A) Chl, (B) *b_bp_*, and (C) CDOM. The reference density layer ρ = 1023.6 is shown (black line). Note that profiles are not evenly spaced in time (see Methods). The times corresponding to SSHA imagery in Fig. 1 are indicated in panel A (white dashed lines) to show where the float was located relative to the LC boundary.
(TIF)

Figure S4 **Comparison of two physical-mixing indicators: the depth of the density layer ρ = 1023.6 and pycnocline depth.** The pycnocline depth was calculated for each profile based on the maximum gradient in density in the upper 300 m of the water column.
(TIF)

Dataset S1 **Float data used in this analysis.** Text file contains the following columns: year, month, day, depth (m), T (°C), S (psu), Chl (µg l^−1^), *b_bp_* (m^−1^), and CDOM (ppb).
(TXT)
