# Supplementary figures and images for: First Autonomous Bio-Optical Profiling Float in the Gulf of Mexico Reveals Dynamic Biogeochemistry in Deep Waters
Source: PLoS One. 2014 Jul 3;9(7):e101658. doi: 10.1371/journal.pone.0101658 (PMC4081586; doi:10.1371/journal.pone.0101658)

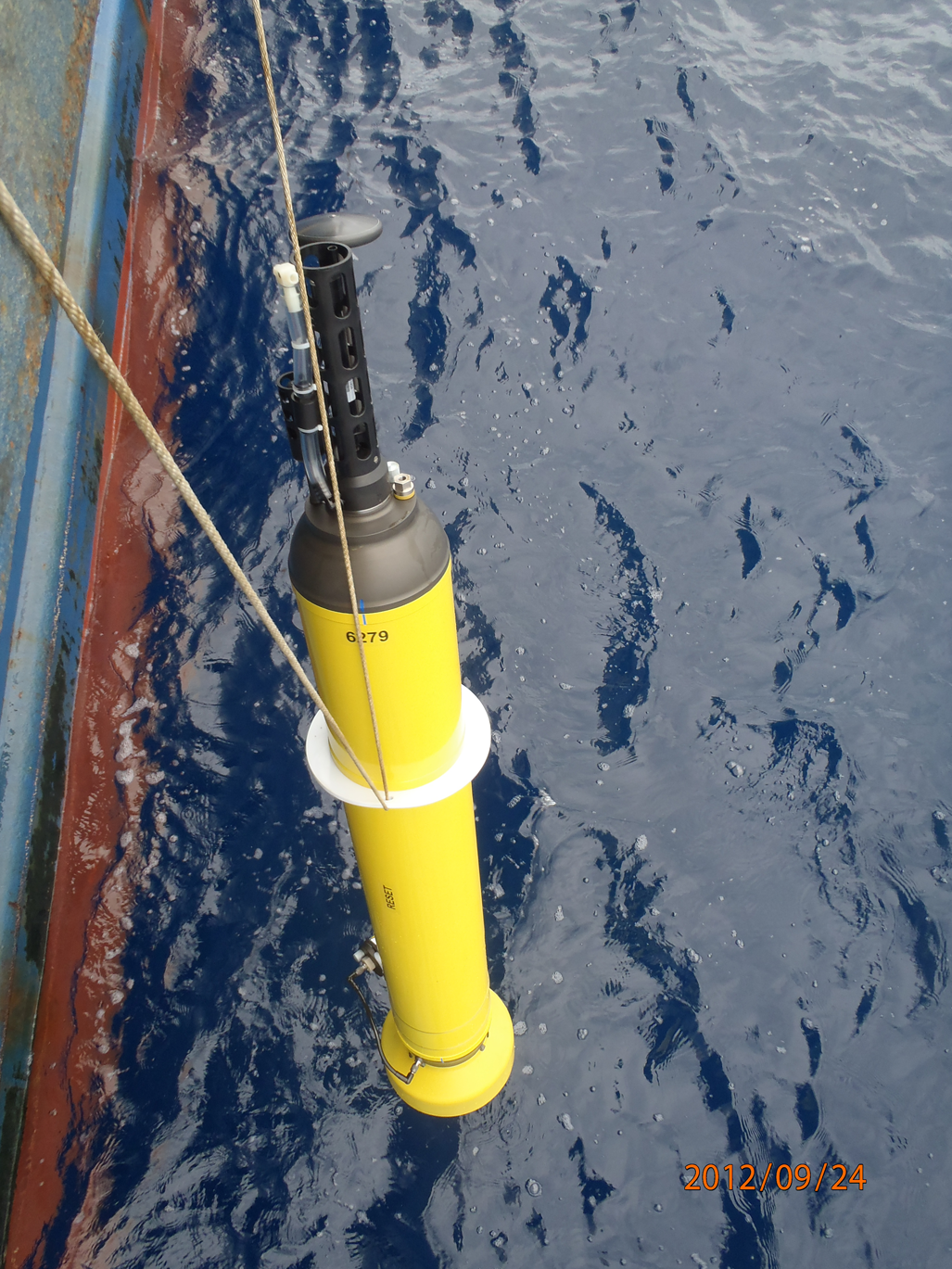

Supplement: Figure S1 — Picture of APEX float being deployed in the Gulf of Mexico. The antenna and pumped CTD are located at the top of the float, whereas the optical sensors are located near the bottom of the instrument (Photo Credit: CANEK group, CICESE). (TIF) [file pone.0101658.s001.tif]

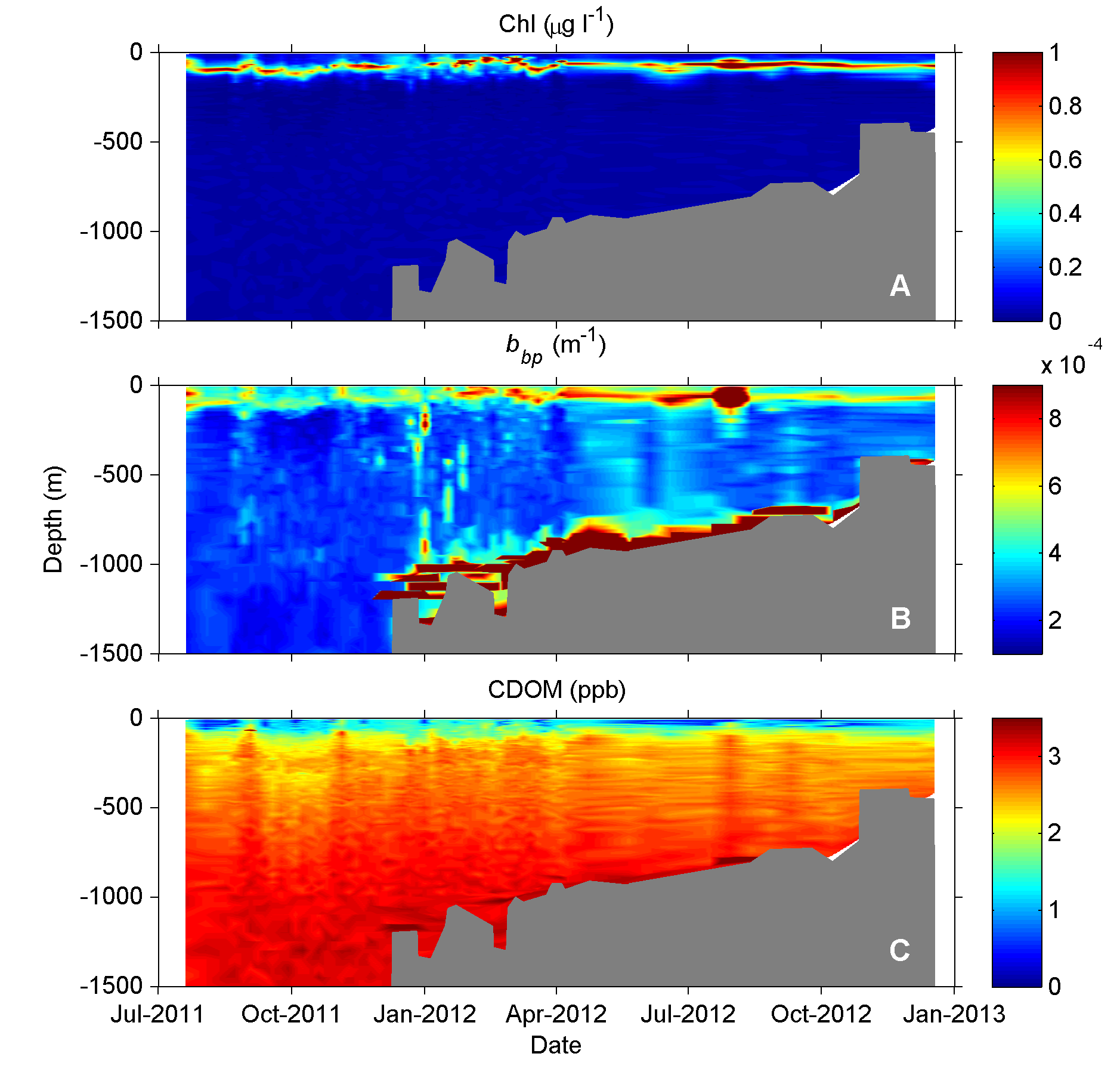

Supplement: Figure S2 — Contour plots of bio-optical profiles over the entire depth range the float transited. (A) Chl, (B) bbp, and (C) CDOM. Bottom depth is shown for reference (shaded grey). Note that profiles are not evenly spaced in time (see Methods). (TIF) [file pone.0101658.s002.tif]

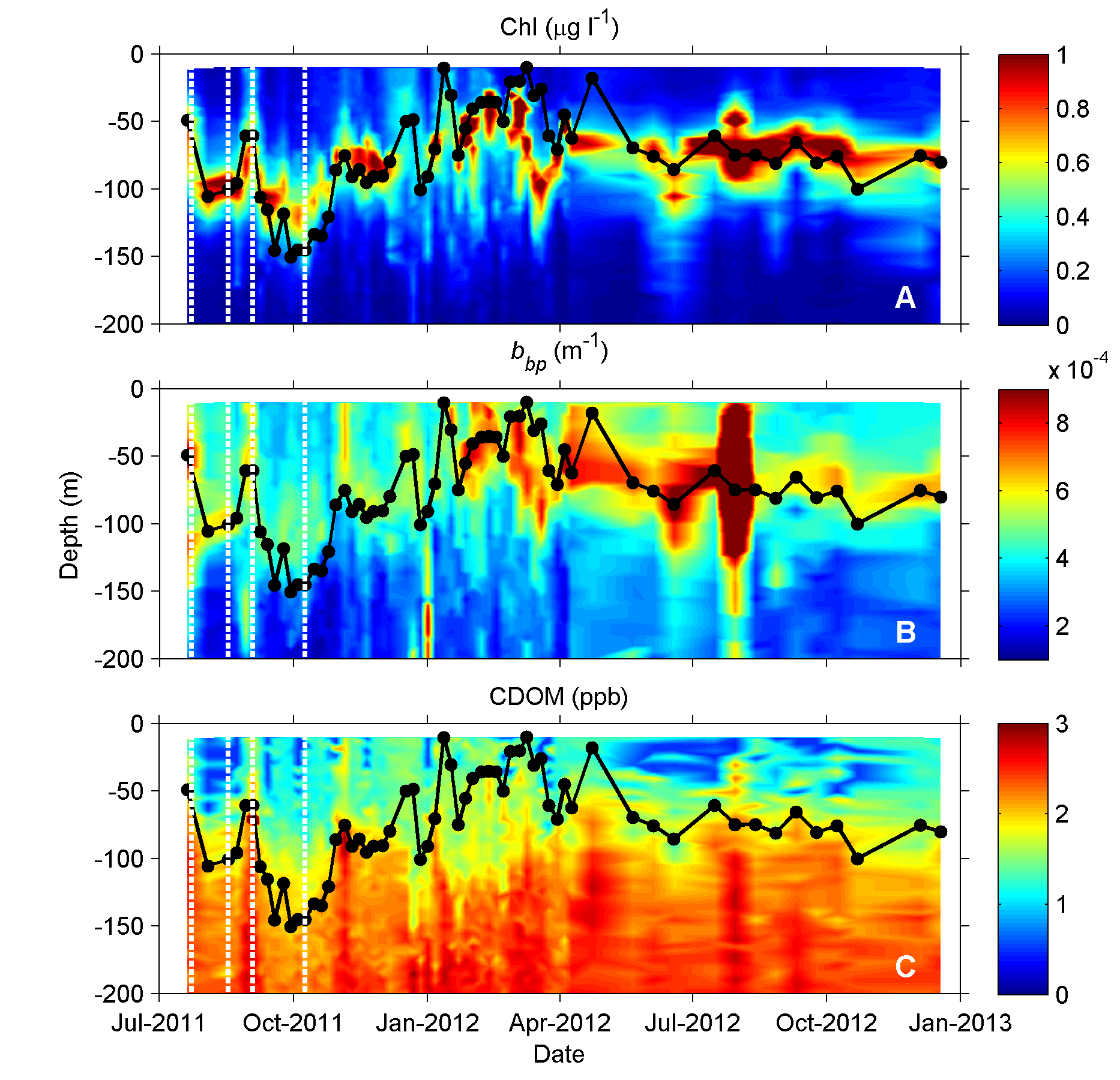

Supplement: Figure S3 — Contour plots of bio-optical profiles for the upper 200 m to emphasize upper-water column dynamics. (A) Chl, (B) bbp, and (C) CDOM. The reference density layer ρ = 1023.6 is shown (black line). Note that profiles are not evenly spaced in time (see Methods). The times corresponding to SSHA imagery in Fig. 1 are indicated in panel A (white dashed lines) to show where the float was located relative to the LC boundary. (TIF) [file pone.0101658.s003.tif]
